# Intestinal mucormycosis in a neonate: A case report and review

**DOI:** 10.4103/0971-9261.71753

**Published:** 2010

**Authors:** Yogesh Kumar Sarin

**Affiliations:** Department of Pediatric Surgery, Maulana Azad Medical College, New Delhi - 110 002, India

**Keywords:** Amphotericin B, mucormycosis, necrotizing enterocolitis, neonate, rhizopus

## Abstract

Mucormycosis is a fungal disease that may rarely invade the gastrointestinal tract of newborn, resulting in high morbidity and mortality. Clinically, it may be indistinguishable from the neonatal necrotizing enterocolitis and the diagnosis is usually made on autopsy or histopathology of excised surgical specimen. We report a neonatal survivor of the illness.

## INTRODUCTION

Mucormycosis is an uncommon, opportunistic fungal infection caused by Mucorales of class Zygomycetes that occurs in immunocompromised human hosts.[1] The first review of neonatal gastrointestinal mucormycosis (GIM) was published in 1994.[2] Of the 22 cases of neonatal GIM reported in English literature till date, 15 have been reported from India.[1,3–6] A survivor of neonatal GIM is reported and the relevant literature is reviewed.

## CASE REPORT

A male neonate (birth weight 2750 g) was born to a 21-year-old primigravida at full term through normal vaginal delivery. Apgar scores of 4 and 10 at 1 and 5 minutes, respectively, were noted. There were no identifiable maternal risk factors. The baby developed respiratory distress soon after birth. Intravenous (IV) antibiotics, ceftriaxone and amikacin, were instituted. Oxygen was administered by hood and a nasogastric tube was placed. At 28 hours of life, his condition deteriorated. He developed septic shock, generalized seizures, electrolyte and acid–base imbalance, azotemia, and required ventilatory support. His condition improved with higher antibiotics (piperacillin + tazobactum and netilmycin) over the next 4 days, wherein ionotropic and ventilatory support were withdrawn. On the 5th day, oral candidiasis was noted and hence IV fluconazole was started. On the 6^th^ day, his condition deteriorated again, wherein he developed abdominal distension, ileus, hemorrhagic nasogastric aspirates and shock, requiring ionotropic support and broad spectrum antibiotics (aztreonam, amikacin and metronidazole). In view of the sudden deterioration, he was referred to us for further management.

**Figure 1:**
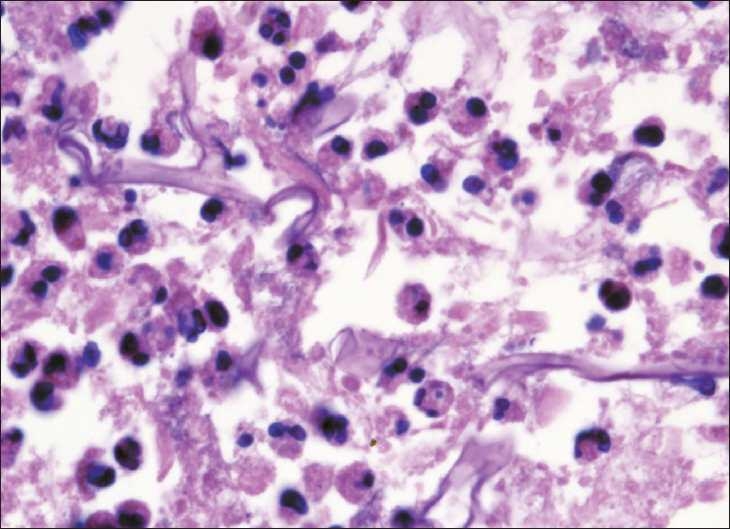
Presence of characteristic aseptate wide hyphae of Mucor within the intestinal segment. (H and E, ×400)

On admission, the general condition was poor and there was a fixed bowel loop felt in the right lower quadrant of the abdomen. Plain abdominal radiographs revealed generalized gaseous distension of the bowel, without any specific radiological features of necrotizing enterocolitis (NEC). The baby was started on meropenem. Since the baby did not show any improvement over the next 2 days, NEC was suspected and he was taken up for laparotomy on day 9 of life. Two circumferential ileal perforations, 16 and 10 cm proximal to ileocecal junction, were noted; the intervening segment was gangrenous. The stomach and colon were normal. The affected ileum was resected and a double-barrel ileostomy was performed. The histopathology of the resected ileum showed mucosal ulceration with presence of multiple epitheloid granulomata admixed with lymphocytes and eosinophils. Broad fungal hyphae with right angle branching were present transmurally, suggesting the diagnosis of intestinal mucormycosis [Figure 1]. The resection margins were free of mucor invasion. The peritoneal fluid and the blood cultures were negative for organisms and fungi. Ileostomy closure was done after 3-week administration of IV liposomal amphotericin B that was well tolerated. The child is thriving well and gaining weight after ileostomy closure.

## DISCUSSION

Mucorales are present in soil, bread, fruit, and decaying material, and hardly show any pathogenicity in normal human host, but they may cause fulminant infection in immunocompromised individuals.[7–9] GIM is the rarest form accounting for 7% of all cases.[10] In childhood and adult GIM, the stomach is the most commonly involved organ, followed by the colon, small bowel and esophagus, whereas in neonatal GIM, the colon is predominantly involved.[5]

Low birth weight (LBW), especially premature, neonates having immature immune system and fragile skin barriers, are at risk of mucormycosis. They are usually treated long in neonatal ICU having an environment of high ambient humidity which may enhance the growth of and exposure to Mucorales, and are administered broad-spectrum intravenous antibiotics and steroids that affect healthy GIT flora.[11] Interventions such as oro-gastric tube placement, endotracheal intubation, and indomethacin therapy are speculated to increase the risk of contracting GIM by causing mucosal injury.[11] Our patient had most of the described risk factors. Other than that, he was neither premature nor of LBW.

GIM has been hypothesized to be a variant of NEC. GIM should be considered in LBW babies with clinical NEC, who have prolonged neutropenia, negligible enteral feedings and treatment with multiple antibiotics. The absence of pneumatosis intestinalis, poor response to usual chemotherapy given for NEC and widespread thrombosis of small vessels of the gut differentiate GIM from classical NEC.[10]

A clinical diagnosis of the condition has never been made.[12] Aggressive early surgery followed by intravenous amphotericin B after histological diagnosis is the mainstay of treatment. Adequate surgical resection reduces the fungal load and the chances of perforation and long-term sequelae like stricture. IV amphotericin B is a toxic drug known to cause multiple organ damage in therapeutic doses. IV liposomal amphotericin has a lesser incidence of nephrotoxicity and is therefore preferred. Rifampicin may be added to amphotericin B.[9] However, survival has been reported in neonatal GIM without the use of amphotericin.[4] This implies that survival also depends upon the extent of disease, the immunological status of patient and the virulence of the organism. The common prophylactic antifungal agents used (such as fluconazole) or even newer agents (voriconazole and caspofungin) are not active against the Mucorales.[9] Oral posaconazole, has been known to be beneficial in refractory cases in adults,[9] but its efficacy and safety in children are yet not established.

The prognosis of neonatal GIM is grave with only seven neonates having survived the illness.[2,4,13,14] The high mortality results from lack of clinical suspicion coupled with inadequate surgery and antifungal therapy. Two of the neonates from our institution, reported previously, had neither received amphotericin B nor survived.[5,6] To conclude, GIM should be considered in LBW neonates with a clinical picture of NEC, who have prolonged neutropenia, negligible enteral feedings and treatment with multiple antibiotics. Aggressive early surgery followed by intravenous amphotericin B after histological diagnosis is the mainstay of treatment.
